# Slow-oscillatory tACS does not modulate human motor cortical response to repeated plasticity paradigms

**DOI:** 10.1007/s00221-022-06462-z

**Published:** 2022-09-29

**Authors:** Claire Bradley, Jessica Elliott, Samuel Dudley, Genevieve A. Kieseker, Jason B. Mattingley, Martin V. Sale

**Affiliations:** 1 Queensland Brain Institute, The University of Queensland, St Lucia, Australia; 2 School of Psychology, Faculty of Health and Behavioural Sciences, The University of Queensland, St Lucia, Australia; 3 Canadian Institute for Advanced Research (CIFAR), Toronto, Canada; 4 School of Health and Rehabilitation Sciences, Faculty of Health and Behavioural Sciences, The University of Queensland, St Lucia, Australia

**Keywords:** Plasticity, Slow-wave oscillations, Transcranial alternating current stimulation, Motor, Sleep, Brain stimulation

## Abstract

Previous history of activity and learning modulates synaptic plasticity and can lead to saturation of synaptic connections. According to the synaptic homeostasis hypothesis, neural oscillations during slow-wave sleep play an important role in restoring plasticity within a functional range. However, it is not known whether slow-wave oscillations—without the concomitant requirement of sleep—play a causal role in human synaptic homeostasis. Here, we aimed to answer this question using transcranial alternating current stimulation (tACS) to induce slow-oscillatory activity in awake human participants. tACS was interleaved between two plasticity-inducing interventions: motor learning, and paired associative stimulation (PAS). The hypothesis tested was that slow-oscillatory tACS would prevent homeostatic interference between motor learning and PAS, and facilitate plasticity from these successive interventions. Thirty-six participants received sham and active fronto-motor tACS in two separate sessions, along with electroencephalography (EEG) recordings, while a further 38 participants received tACS through a control montage. Motor evoked potentials (MEPs) were recorded throughout the session to quantify plasticity changes after the different interventions, and the data were analysed with Bayesian statistics. As expected, there was converging evidence that motor training led to excitatory plasticity. Importantly, we found moderate evidence against an effect of active tACS in restoring PAS plasticity, and no evidence of lasting entrainment of slow oscillations in the EEG. This suggests that, under the conditions tested here, slow-oscillatory tACS does not modulate synaptic homeostasis in the motor system of awake humans.

**Supplementary Information:**

The online version contains supplementary material available at 10.1007/s00221-022-06462-z.

## Introduction

Synaptic plasticity–the modulation of synaptic neural transmission strength–is a prominent biological mechanism underlying learning (Moser 1998; Rioult-Pedotti 2000; Hodgson et al. 2005). Importantly, the likelihood that a synapse will undergo plastic change is not constant over time, but depends on previous history of synaptic activity and plasticity–a phenomenon known as ‘meta-plasticity’ (Abraham and Bear 1996). Reflective of this, interventions known to trigger plasticity in the human motor system interfere with, and even reverse, each other’s effects when applied sequentially (Shadmehr and Brashers-Krug 1997; Ziemann et al. 2004; Ziemann and Siebner 2008), highlighting the importance of previous history of network activity for plasticity effects.

Sleep is a major physiological modulator of learning and plasticity (Maquet 2001; Diekelmann and Born 2010). Whether for a whole night or a short nap, sleep has been shown to enhance retention of learned material (Tucker et al. 2006; Mascetti et al. 2013) and allows further acquisition (Mander et al. 2011; Antonenko et al. 2013). At a synaptic level, sleep has been hypothesized to achieve a general downscaling of synaptic weights (Tononi and Cirelli 2014). This account of sleep function–the synaptic homeostasis hypothesis–posits that synapse modifications accumulate during the waking day due to learning and non-specific exposure to environmental stimuli. The resulting increased synaptic strength imposes metabolic and computational pressure, which is resolved through re-normalization of synaptic strength during sleep. Although experimental studies testing this hypothesis in humans are scarce, it appears sleep may recalibrate cortical excitability (Huber et al. 2013), synaptic plasticity (Kuhn et al. 2016), and learning (Fattinger et al. 2017) in healthy adults.

Slow-wave oscillations—low-frequency oscillatory electrical activity characteristic of non-rapid eye movement sleep—are a prime candidate mechanism for synaptic strength downscaling. Slow-wave oscillations increase after a learning episode (Molle et al. 2004) and with time awake, whereas they decrease during sleep as the night progresses (Tononi and Cirelli 2014). Boosting their amplitude and duration during sleep by means of non-invasive brain stimulation results in better encoding of information and increased memory retention (Marshall et al. 2006; Antonenko et al. 2013; Ladenbauer et al. 2016, 2017), whereas disrupting them impairs the restorative property of sleep (Aeschbach et al. 2008; Fattinger et al. 2017, although see Paßmann et al. 2016). Interestingly, a causal link between slow-wave oscillations and learning is further suggested by the finding that these oscillations may be entrained in the *awake* human brain and that such entrainment can enhance encoding processes (Kirov et al. 2009). However, whether slow-wave oscillations – without the concomitant requirement of sleep–play a causal role in synaptic homeostasis has never been investigated in humans.

Here, we investigated whether slow-oscillatory activity, in line with its hypothesized synaptic downscaling function, enables successive plasticity interventions to exert their full effects. We used two plasticity-inducing protocols: motor training (MT) and excitatory paired associative stimulation (PAS). MT involves repeating a ballistic finger movement over the course of 30 min to one hour, resulting in faster movement post-training and increased excitability of the motor tract. While motor skill learning involves a distributed network of sub-cortical and cortical areas, this particular paradigm relies on plasticity of the primary motor cortex in the early phase of consolidation (Muellbacher et al. 2001, 2002). PAS, on the other hand, involves passive stimulation of a peripheral nerve, rhythmically combined with transcranial magnetic stimulation (TMS) of the motor cortex. Its effects are compatible with synaptic plasticity mechanisms within motor cortex. The specific timing between peripheral and central stimulation determines whether these plastic changes are excitatory or inhibitory (Stefan et al. 2000; Wolters et al. 2003). Importantly, both these plasticity-inducing protocols (MT and PAS) have been shown to involve the motor cortex, are thought to rely on synaptic plasticity mechanisms, and have been shown to interfere with each other in a homeostatic way (Ziemann et al. 2004): specifically, excitatory plasticity caused by MT prevents excitatory PAS-plasticity effects from being expressed. We delivered slow-oscillatory transcranial alternating current stimulation (tACS) – an intervention shown to entrain or induce oscillatory brain activity (Kirov et al. 2009)—between MT and PAS to test whether inducing slow-oscillatory activity would prevent homeostatic interference between the two plasticity paradigms. Motor cortical plasticity was quantified indirectly by measuring the amplitude of transcranial magnetic stimulation (TMS) evoked motor-evoked potentials (MEPs). We hypothesized that following active tACS, and in spite of the previous motor training, PAS would result in an increase in MEPs, consistent with a synaptic downscaling effect of slow oscillations as predicted by the synaptic homeostasis hypothesis. We also hypothesised that this effect would not be seen after sham (inactive) or control tACS not targeting the motor network. We used Bayesian statistics to quantify the extent of evidence for or against our various hypotheses. This approach is preferable to null-hypothesis significance testing, in that it can distinguish between situations where there is evidence for the null hypothesis, for the alternative hypothesis or not enough evidence to distinguish between both. This can be particularly useful in interpreting the absence of difference between two conditions but applies more generally to all inferences (Biel and Friedrich 2018; Dienes and Mclatchie 2018).

## Materials and methods

### Participants

Seventy-eight participants (mean age ± SD: 24 ± 5 years; 50 women; split into two groups) took part in the study. All participants were right-handed (mean laterality quotient = 0.92, range 0.5–1.00) as assessed by the Edinburgh Handedness Questionnaire (Oldfield 1971). All participants gave written informed consent prior to participation in the study, which was approved by The University of Queensland Human Research Ethics Committee. All research was performed in accordance with relevant guidelines and regulations. Participants were screened for family history of epilepsy, consumption of neuroactive drugs, and history of neurosurgery or brain injury, using a TMS safety questionnaire (Keel et al. 2001). Four participants were excluded from the final sample due to non-compliance with motor training (*n* = 1), a request to exit the study before the end of the last session (*n* = 1), very small MEPs (< 0.1 mV, *n* = 1), and tACS equipment failure (*n* = 1). Final participant numbers were as follows. Active vs. sham tACS condition: *n* = 36, mean age ± SD: 24 ± 4 years; 22 women; mean laterality quotient = 0.94. Control tACS montage condition: *n* = 38, mean age ± SD: 24 ± 5 years; 28 women; mean laterality quotient = 0.89.

### Overview of experimental procedure

**Fig. 1:**
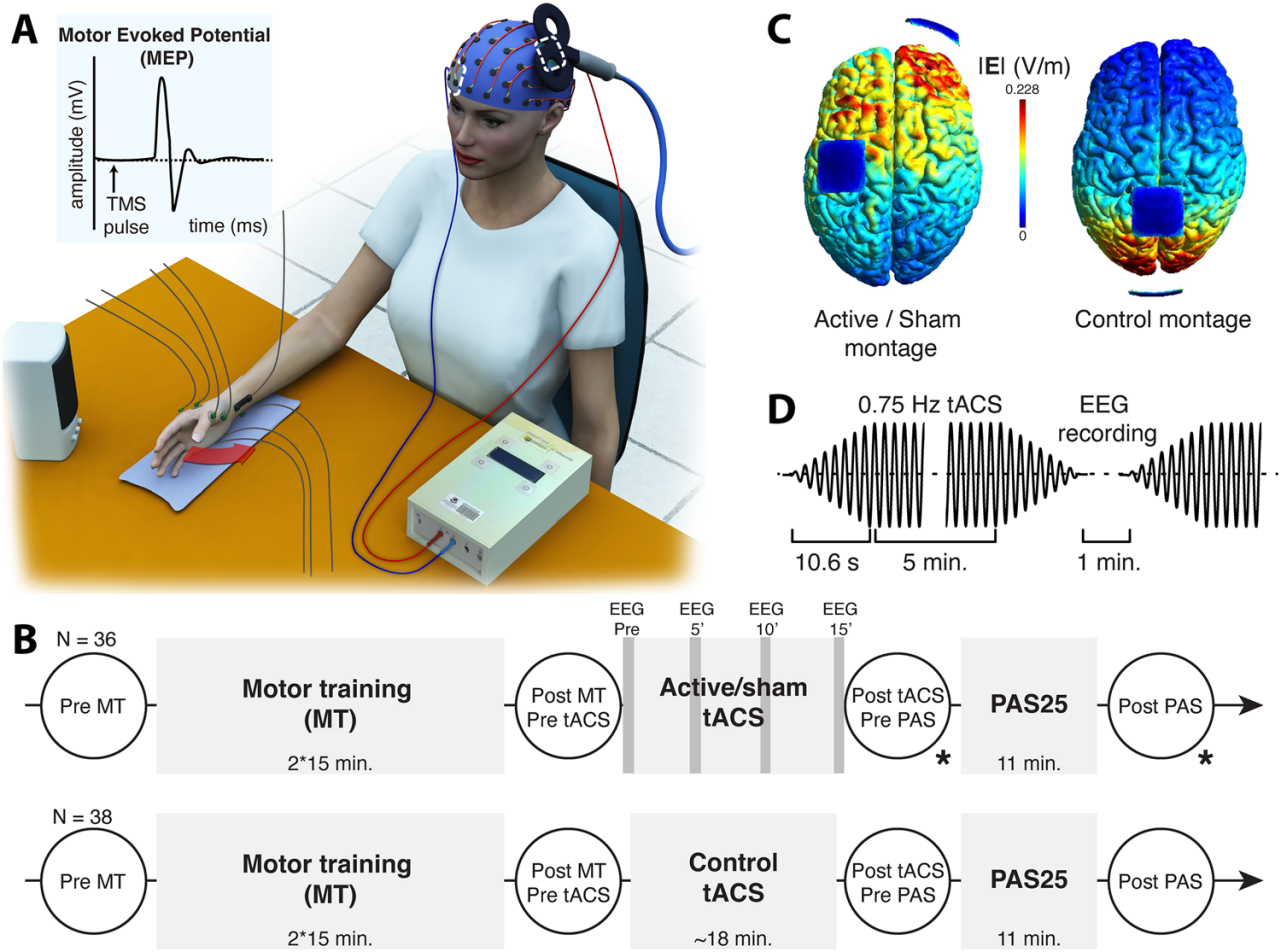
Experimental setup and study protocol. **A** In the active vs. sham condition, transcranial magnetic stimulation (TMS) was combined with electromyography to record motor-evoked potentials (MEPs, i.e. the muscle response to a TMS pulse, *blue inset*) from right *abductor pollicis brevis* (APB) and two other intrinsic hand muscles (first dorsal interosseous, FDI and *abductor digiti minimi*, ADM). The TMS coil was positioned over left primary motor cortex, on top of an electroencephalography (EEG) cap (used to record electrical brain activity) and a transcranial alternating current stimulation (tACS) electrode (used to deliver electrical stimulation, *dashed white lines*). The return electrode for tACS was placed over the contralateral supra-orbit. A peripheral nerve stimulator was placed on the right wrist of participants to allow median nerve stimulation for the paired associative stimulation protocol. An accelerometer was taped to the participant’s right thumb to measure thumb speed during motor training (*red arrow*, accelerometer not shown here). Setup was identical in the control tACS condition, except that a different tACS montage was used and there was no recording of FDI, ADM or EEG. **B** Session timeline of MEP recordings (white circles) and three successive experimental manipulations (grey rectangles), for the active and sham tACS condition (top, *N* = 36, two sessions per participant) and the control tACS condition (bottom, *N* = 38, one session). *MT* motor training, *tACS* transcranial alternating current stimulation, *PAS*: paired associative stimulation. Darker grey bars denote periods selected for EEG analysis before, during and after tACS (1 min for each EEG segment, 3 5-min segments of tACS, see below); *denotes additional MEPs collected at an adjusted stimulation intensity, in a subset of participants. **C** Cortical grey matter projection of the norm of the electric field (V/m) generated by the active vs. sham montage (left) and the control montage (right). Dark blue squares represent the two tACS electrodes. Modelling and visualization were performed in SimNIBS (Thielscher et al. 2015). **D** Summary stimulus waveform for one out of three segments of tACS stimulation. In the active vs. sham condition, resting EEG was recorded continuously from 1 min before the start of tACS to one minute after the end of tACS, allowing extraction of 1-min periods prior to and immediately following each tACS segment. Sham stimulation only consisted of the ramp-up and ramp-down, after which stimulation was turned off (total stimulation time: 63.6 s)

In the active vs. sham tACS condition, participants attended two experimental sessions, separated by at least one week. After general experimental setup, the cortical motor hotspot was located on the scalp, and motor and peripheral electrical thresholds were measured, as described in detail below. Participants then performed a motor training (MT) task for 30 min (Fig. 1A, B), followed by tACS delivered over an 18-min interval (fronto-motor montage), after which a further period of plasticity was induced in the motor cortex using TMS and concurrent stimulation of the median nerve (PAS protocol). MEPs and resting EEG recordings were collected before motor training, before and during tACS, as well as before and after PAS-plasticity induction. Resting motor threshold was re-evaluated after PAS to investigate possible cellular/intrinsic plasticity effects (Delvendahl et al. 2012). The two sessions were identical except that in one of them, sham (placebo) tACS was delivered instead of active stimulation. Delivery of sham and active tACS was randomized and counter-balanced across participants. tACS delivery was double-blinded: participants were not told whether they received active or sham tACS in a given session and the experimenter who programmed the tACS and monitored the EEG was different from the experimenter collecting MEPs and analysing them. For each participant, the two experiments were performed at the same time of day to minimise response variability due to circadian factors (Sale et al. 2007, 2008). A similar procedure was followed in the control tACS montage condition, as described in detail below.

### Neuro-navigated transcranial magnetic stimulation (TMS) of left motor cortex

Monophasic TMS pulses were applied through a figure-of-eight coil (outer diameter of each wing 70 mm) connected to a Magstim 200^2^ magnetic stimulator (Magstim, UK). The coil was held tangentially to the skull with the handle pointing backwards and laterally at an angle of 45° to the sagittal plane, at the optimal scalp site to evoke an MEP in the relaxed *abductor pollicis brevis* (APB) muscle of the right hand. With this coil placement, current flow was induced in a posterior-to-anterior direction in the brain. A neuro-navigation system (Visor2, ANT Neuro, The Netherlands) that tracks the position of the coil relative to the participant’s head ensured consistent placement of the TMS coil throughout the entire session (ensuring deviations of no more than 5 mm and 5° error in position and angle).

### Assessing plasticity in the cortico-spinal tract: motor-evoked potentials (MEPs)

Participants were seated comfortably in an experimental chair with their arms resting on a table and their eyes open, gazing at a fixation cross on a screen in front of them (Fig. 1A). Surface electromyographic (EMG) recordings from the APB, first dorsal interosseous (FDI) and *abductor digiti minimi* (ADM) muscles of the right hand were obtained using bipolar Ag–AgCl electrodes in a belly-tendon montage. EMG signals were amplified 1000 times, filtered (20 Hz–2000 Hz, 50 Hz notch filter) via a NeuroLog system (Digitimer, UK), digitized online with a data acquisition interface and Signal software (CED, Cambridge Electronic Design, UK) and stored on computer for offline analysis. The EMG signal from the APB muscle was continuously monitored on an oscilloscope throughout the session to ensure that the muscle was relaxed (absence of background activity, which would otherwise bias MEP measurements). If background activity was detected, participants were reminded to relax their hand and trials with background activity were discarded.

Resting motor threshold was defined as the intensity eliciting MEPs above 50 μV in 5 out of 10 consecutive trials (Rossini et al. 1994). Resting motor threshold was assessed at baseline and once again just after PAS. Mean threshold ± SD at baseline and after PAS was 52% ± 8% and 52.5% ± 8% of maximum stimulator output in the active session, and 52% ± 9% and 52% ± 10% in the sham tACS session. Test intensity was set at 130% of resting motor threshold. Cortico-spinal excitability was assessed at several time points during each experimental session: before motor training, 5 min after motor training (e.g. immediately before tACS), immediately after tACS and 5 min after PAS. Mean peak-to-peak amplitude of the APB MEP at rest was calculated by averaging the individual peak-to-peak amplitudes of MEPs elicited by 21 separate TMS pulses, delivered at ~ 0.2 Hz. The first trial was systematically discarded to avoid startle responses, while subsequent trials were discarded if they displayed activity exceeding the range of background EMG at rest during the 500 ms preceding a TMS pulse. Peak-to-peak MEP amplitude was detected in the window 6.8–136.5 ms following a TMS pulse. In a subset of participants (*n* = 29), TMS stimulation intensity post-tACS (pre-PAS) was adjusted so that MEPs had an amplitude that matched baseline MEPs (pre-motor training). If this amplitude adjustment took more than a few minutes, the second MEP block was recorded at the standard intensity (130% of resting motor threshold), so the timing of MEP assessments was not jeopardised. This intensity-adjusted MEP measure was taken again after PAS, in a counter-balanced order with the original intensity.

### Motor training

The motor training task was adapted from Muellbacher et al. (Muellbacher et al. 2001) and Ziemann et al. (Ziemann et al. 2004). Participants’ right arm rested on a table, bent 90° at the elbow and attached to a guide to ensure consistent initial position for the movement. Participants were requested to repeatedly perform a fastest possible thumb abduction movement of the right hand (thumb ‘flick’). Abductions were paced by a tone at a rate of 0.5 Hz for 30 min (2*15 min with one minute break). After a brief familiarisation with the task and movement, acceleration was measured using a custom-made accelerometer mounted on the thumb; trial-by-trial acceleration in the horizontal plane was displayed on a monitor and participants were repeatedly encouraged to maximize this component. The peak-to-peak amplitude of acceleration in the horizontal plane for the first 20 and the last 20 trials (excluding familiarisation period) were averaged to quantify performance change following motor training.

### Slow-oscillatory transcranial alternating current stimulation (tACS)

tACS was administered via a NeuroConn stimulator (neuroCare Group GmbH, Germany) with two 4 × 4 cm electrodes. One electrode was positioned over the motor cortical hotspot, the other electrode was placed over the right supraorbital region (Fig. 1C). This montage was chosen because it has repeatedly been shown to effectively modulate MEP amplitudes when used to deliver transcranial direct current stimulation (tDCS) (Nitsche and Paulus 2000, 2001; see Dissanayaka et al. 2017 for a review). tDCS and tACS differ in their temporal profile and mode of action, but not in the spatial distribution of induced electric fields. While the e-field modelling does not focus primarily on M1 (Fig. 1C), the chosen montage has a history of successfully modulating cortical elements that influence MEP amplitude – which was used as the primary outcome measure for this study. Electrodes were held in place by conductive paste (Ten20 EEG paste, Weaver and company, USA). Using electrode paste rather than saline solution for concurrent tES-EEG is recommended (Antal et al. 2017) and yields several advantages: (1) homogeneous contact over the entire electrode, providing a well-defined interface; (2) no drying out, providing stable impedance over time, (3) strong tethering to the scalp, providing consistent positioning over time, (4) no dripping, preventing bridging with EEG electrodes that are placed away from the tACS electrode.

tACS was applied at a frequency of 0.75 Hz and a stimulus intensity of 1 mA peak-to-peak, resulting in a peak current density of 0.031 mA/cm^2^, which is within safety limits (Antal et al. 2017). Stimulation was applied over 3 × five-minute blocks, each separated by 1 min, to allow for EEG recording, and in keeping with (Marshall et al. 2006) and (Kirov et al. 2009). Each stimulation block consisted of 225 cycles (300 s) and included a ramp up and ramp-down period of 8 cycles (10.6 s) (Fig. 1D). Impedance was monitored throughout stimulation and the stimulation was aborted if impedance exceeded 20 kOhm. For the sham condition, the stimulus parameters were the same except that following the 8-cycle ramp-up and ramp-down, the stimulator was turned off. Participants were not informed about the type of stimulation they were receiving; they sat in silence with their eyes open.

### Paired associative stimulation (PAS)

The PAS paradigm involved a series of paired peripheral and cortical stimuli (Stefan et al. 2000; Wolters et al. 2003; Ziemann et al. 2004). A peripheral electrical stimulus was delivered to the median nerve of the right wrist at an intensity that evoked a clear response in the APB muscle of at least 0.2 mV amplitude (Active vs. sham tACS condition: mean ± SD perceptual threshold: 3.17 ± 0.8 mA, stimulation intensity: 7.1 ± 2.3 mA; Control tACS montage condition: mean ± SD perceptual threshold: 2 ± 0.8 mA, stimulation intensity: 7.4 ± 2.5 mA), using a constant current stimulator (DS7 stimulator; Digitimer, UK) with bipolar surface electrodes separated by 30 mm (cathode proximal). Stimuli were square waves with a pulse width of 200 μs. Each electrical stimulus was followed by suprathreshold TMS (intensity: 130% of resting motor threshold) over the hand area of the contralateral (left) motor cortex, with a 25 ms delay. A total of 132 paired peripheral and cortical stimuli were delivered at an average frequency of 0.2 Hz. Because spatial attention has been shown to enhance the effects of PAS (Kamke et al. 2014), participants were asked to overtly monitor a blinking LED strapped to their right thumb for infrequent changes in the rhythm of blinking and to report the number of events at the end of the PAS stimulation.

### Electroencephalography (EEG)

To investigate potential entrainment of endogenous slow oscillations by active tACS (Kirov et al. 2009), 64-channel EEG was recorded at rest with eyes open (active vs. sham conditions only). EEG could not be collected in three participants due to the EEG cap not fitting (*n* = 1), not enough time to apply the cap in one session (*n* = 1) and motor threshold being too high with the cap on (*n* = 1).

### EEG recording

Spontaneous EEG was recorded with a TMS-compatible 64-channel EEG cap (BrainCap, BrainProducts, Germany; Ag/AgCl electrodes) in accordance with the 10–10 extended international system. All electrodes were referred to the right mastoid and impedance was kept below 5 kΩ using a viscous electrode paste (Abralyt HiCl Gel, EasyCap, Germany); the ground electrode was incorporated in the cap at AFz. BrainRecorder software and BrainAmp MR Plus amplifiers (BrainProducts, Germany) with a 5000 Hz sampling rate and low-pass filter of 1000 Hz (no high-pass filter, resulting in a DC recording) were used to record periods of EEG throughout the experiment. Participants were at rest, with eyes open and gaze steadied on a fixation point.

### EEG analysis

Four segments of 1-min resting EEG were extracted from the continuous recordings: one before tACS (“Pre”) and one after each 5-min block of tACS stimulation (“5’”, “10’” and “15’”), using BrainVision Analyser (Brain Products, Germany). Special care was taken that segments started no later than 10 ms after the end of the active stimulation (as evidenced by a clear artefact); segments in the sham condition were taken at the same time points as in the active condition. Some electrodes presented an exponential decaying artefact following active tACS termination in most participants which could contaminate estimation of power at low frequencies (Woods et al. 2016); these electrodes were discarded from further analysis (9 electrodes in total, all located near or on the tACS stimulation pads: FC3, FC1, C3, C1, CP1, CP3, Fp2, AF4, AF8), as were TP9 and TP10 (systematically noisy) and Fp1 and Fpz (because of TMS neuro-navigation markers). Importantly, slow-wave oscillations are known to have a widespread spatial distribution on the scalp (see e.g. Huber et al. 2008; Fattinger et al. 2017), and previous reports showing modulation of slow-wave activity by tACS (Kirov et al. 2009; Marshall et al. 2006, 2011) had relatively low EEG spatial resolution (between 7 and 11 electrodes in total), such that the changes that are described as ‘local’ may be relatively widespread. The remaining scalp electrodes in our montage were therefore considered sufficient in number and location to detect potential slow-wave activity frequency-specific entrainment of EEG near tACS electrode locations, as well as widespread scalp changes in oscillatory activity outside of the frequency of stimulation, as reported in previous research (Kirov et al. 2009; Marshall et al. 2006, 2011).

Data were imported in EEGLAB v13.6.5b (Delorme and Makeig 2004) running in Matlab R2016a, segmented, down-sampled to 1000 Hz and filtered (notch: 50 Hz, low-pass: 100 Hz). Periods of noise (visual inspection for speech, movements) were discarded; similarly, noisy or flat electrodes were interpolated. ICA analysis (extended binICA algorithm) was performed on filtered (band-pass: 2–35 Hz), PCA-treated data (to compensate for rank-reduction induced by interpolating electrodes), to identify and reject vertical and horizontal eye movement artefacts (Chaumon et al. 2015). The correction (ICA weights and sphering matrix) was then applied to the less-filtered data that were considered for further analysis. The power spectrum density was calculated using the ‘spectopo’ function of EEGLAB (average of FFT of 5 s segments, Hanning-window, 50% overlap). Average power values were taken over 6 contiguous frequency bands (as in (Kirov et al. 2009)): tACS-frequency: [0.5–1 Hz], delta: [1–4 Hz], theta: [4-8 Hz], low alpha: [8–12 Hz], high alpha: [12–15 Hz] and beta: [15–25 Hz]. Values were further averaged over the three time points post-tACS and decibel-change values relative to baseline (pre-tACS) were computed.

### EEG statistical analysis

Of the thirty-three participants with EEG recordings, eight participants were excluded due to poor data quality as assessed during the artefact rejection phase, resulting in a final sample of *n* = 25 participants. To investigate whole-scalp changes in power, cluster-based permutation testing was performed in FieldTrip (Oostenveld et al. 2011) (sample statistic: dependent samples t-test; test statistic: maximum of sum of *t*-values, MonteCarlo method, 1000 permutations, cluster-forming alpha level: 0.05, two-tailed test alpha level: 0.05) to compare: (a) baseline maps before active and sham tACS, (b) post-tACS to pre-tACS (active and sham separately), (c) decibel (dB)-change post-active tACS to post-sham tACS. Signal from the 10 electrodes surrounding the motor tACS pad (FCz, Cz, CPz, CP5, C5, FC5, F3, F1, P1, P3) was pooled to extract mean power values in the tACS frequency over all time points (Pre, Post 5’, Post 10’ and Post 15’ tACS). These were analysed in GraphPad Prism7, by means of a two-way repeated-measures ANOVA, with factors Time (Pre, 5’, 10’ and 15’) and tACS type (Active and Sham), followed by a post-hoc Dunnett’s multiple comparisons test, comparing all later time points to baseline (‘Pre’).

### Control tACS condition: posterior midline montage

Participants in the control tACS condition underwent procedures identical to those described above, except for the following differences. Participants attended only one experimental session, with no EEG recording. MEPs were recorded from the APB muscle only. Resting motor threshold was defined as the intensity eliciting MEPs above 50 μV in 10 out of 20 consecutive trials (Rossini et al. 2015), and MEPs were calculated from the average of 41 separate pulses instead of 21. Finally, intensity was not adjusted to 1 mV before PAS and motor threshold was not re-measured after PAS, resulting in a comparable number of pulses delivered throughout the session in all groups. Mean resting motor threshold ± SD at baseline was 46% ± 10% of maximum stimulator output. Importantly, control (active) tACS was delivered through a posterior midline montage (Oz-CPz, see Fig. 1D for an electric field distribution). This montage was chosen to minimize overlap between its electric field and that of the fronto-motor active montage, and with areas supporting motor learning in general (including the cerebellum).

### Statistical analysis

Statistical analyses of MEPs were conducted in JASP (JASP Team (2020), n.d.) (Version 0.14.1). Data analysed were acceleration and raw MEP amplitude (as recommended by Lahr et al. (2016)). In the active vs sham condition (*n* = 36), Bayesian two-way repeated-measures ANOVAs, with factors ‘time’ (‘Pre’ and ‘Post’ intervention) and ‘tACS type’ (‘active’ or ‘sham’ tACS) were used to analyse acceleration and MEP amplitude data. Four ANOVAs were conducted: one to examine the effect of motor training and tACS type on acceleration data, and three separate ANOVAs to examine the effect of the three interventions (motor training, tACS and PAS) and tACS type on MEP amplitude. Post-hoc Bayesian paired t-tests were used to explore specific contrasts of interest. In the control tACS montage condition (*N* = 38), Bayesian paired t-tests were used to examine the effect of time (‘Post’ – ‘Pre’ intervention). Four tests were conducted: one to investigate the effects of motor training on acceleration, and one to investigate each of the effects of motor training, tACS and PAS on MEP amplitude. We used Cauchy priors, centred on zero, with default values in JASP; these priors assume a higher likelihood of small effect sizes relative to large effect sizes. In these analyses, the data are used to gather evidence for or against the null hypothesis (H_0_: no difference between measurements) or the alternative hypothesis (H_1_: existence of a difference between measurements (Wagenmakers et al. 2018)), which is expressed using a Bayes factor. We report BF_10_, to quantify evidence favouring the alternative hypothesis over the null, for t-tests, and BF_incl_, the Bayes factor for inclusion of an effect, calculated across all models containing that effect within an ANOVA model. Effect sizes are reported as partial eta-squared (for ANOVA effects) or as Cohen’s d (for t-tests). We adopted the following labelling convention: BF_10_ > 10 indicates strong support for H_1_ over H_0_; BF_10_ > 3 indicates moderate support for H_1_ over H_0_; 1 < BF < 3 indicates anecdotal support for H_1_ over H_0_; 1/3 < BF_10_ < 1 indicates anecdotal support for H_0_ over H_1_; 1/10 < BF_10_ < 1/3 indicates moderate support for H_0_ over H_1_; BF_10_ < 1/10 indicates strong evidence for H_0_ over H_1_; and BF_10_ = 1 indicates no evidence for H_0_ or H_1_.

## Results

**Fig. 2:**
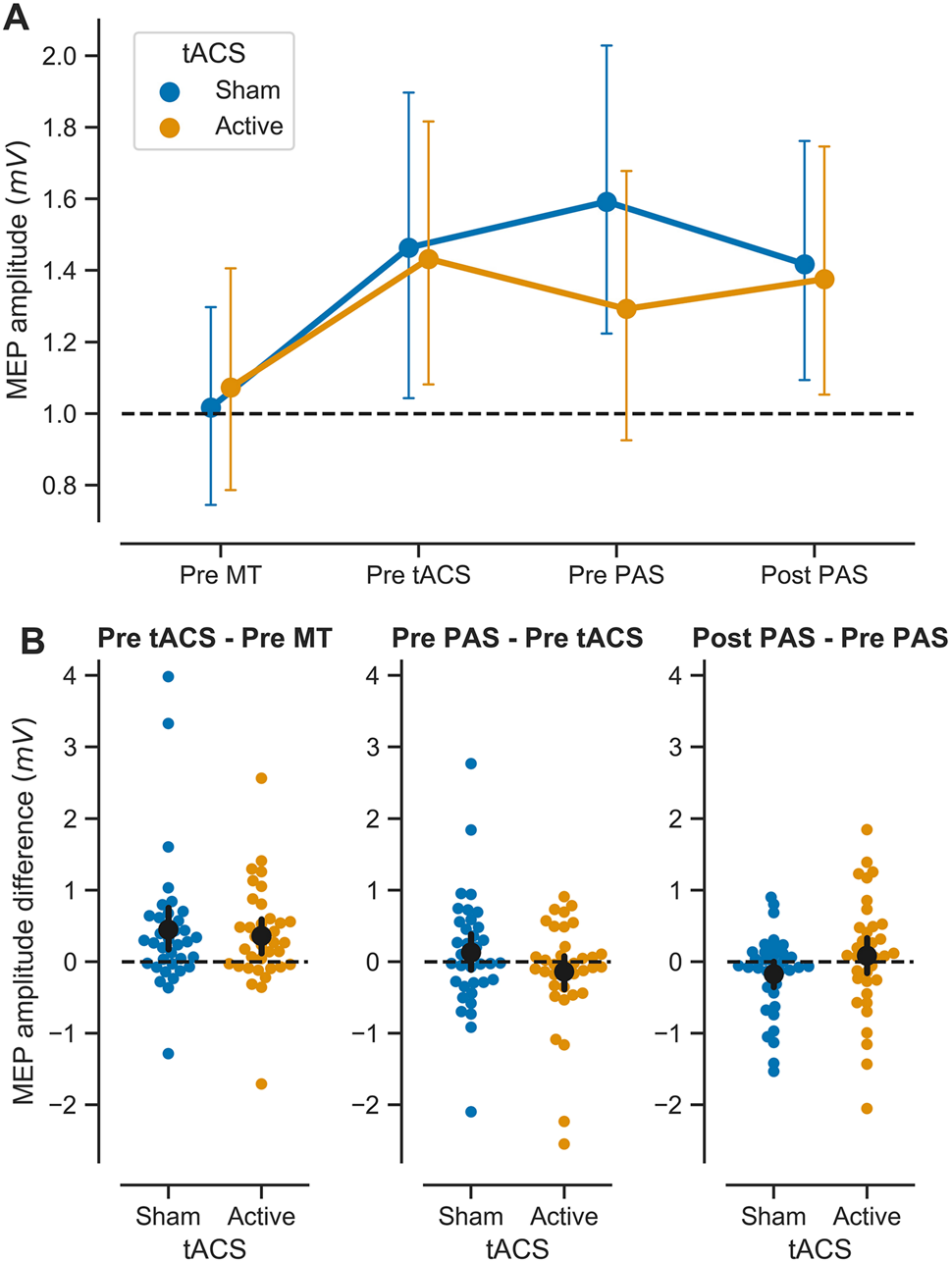
Effect of sham and active tACS on MEP amplitude in response to successive plasticity paradigms. **A** Raw MEP amplitude (mV) at four experimental time points: at baseline (Pre MT), after motor training/before tACS (Pre tACS), after tACS/before PAS (Pre PAS) and after PAS (Post PAS), in the sham (blue) and active (orange) tACS sessions. Symbols represent the mean; error bars denote 95% confidence intervals (CI, 1000 bootstrap iterations). Dotted line denotes baseline MEP amplitude. **B** Mean MEP amplitude difference over each intervention (Post – Pre, black symbol), with 95% CI (black bar) and individual observations (coloured symbols). From left to right: Effect of 30 min of ballistic motor training (MT) on MEP amplitude (left); note that this graph presents data collected in active and sham tACS sessions *before* applying any brain stimulation intervention. Effect of 18 min. of slow-oscillatory transcranial alternating current stimulation (tACS) (middle). Effect of paired associative stimulation (PAS_25_, excitatory protocol) on MEP amplitude (right). Dotted line denotes null difference between time points. *N* = 36

*Motor training induces robust increases in performance and MEPs.* In the first phase of each session, participants performed 30 min of ballistic thumb abduction. We hypothesised that motor training would increase acceleration and MEP amplitudes from the *abductor pollicis brevis* (APB) in all conditions. In the active and sham tACS sessions, before any application of tACS, acceleration was higher after motor training than at baseline (Table 1). A Bayesian two-way repeated-measures ANOVA on horizontal thumb acceleration before and after motor training revealed strong evidence for a main effect of time (BF_incl_ = ∞, η_p_^2^ = 0.74), with moderate evidence against a main effect of tACS type (please note that at this stage, no tACS has been delivered; this test simply compares sessions at baseline and after motor training) (BF_incl_ = 0.2, η_p_^2^ = 0.008) and moderate evidence against their interaction (BF_incl_ = 0.3, η_p_^2^ = 0.056). This shows that baseline performance was not different for the two tACS sessions within the group. Consistent with our hypothesis and with previous results using the same or similar training paradigms, motor training resulted in an increase in MEP amplitude (Fig. 2A, B left panel). A Bayesian two-way repeated-measures ANOVA of MEP amplitude revealed strong evidence for a main effect of motor training (BF_incl_ = 879.2, η_p_^2^ = 0.249), with moderate evidence against a main effect of tACS type (BF_incl_ = 0.14, η_p_^2^ = 8.55 × e^−4^) and moderate evidence against their interaction (BF_incl_ = 0.15, η_p_^2^ = 0.012), reflecting larger MEP amplitudes after motor training than at baseline. Again, this confirms that MEPs and their modulation by motor training were not different at baseline.

**Table 1:** Acceleration (arbitrary units). Mean ± SD

	*N*	Pre MT	Post MT
Active tACS	36	1.51 ± 0.7	2.32 ± 0.7
Sham tACS	36	1.36 ± 0.63	2.37 ± 0.56
Control tACS	38	1.44 ± 0.55	1.95 ± 0.53

**Fig. 3:**
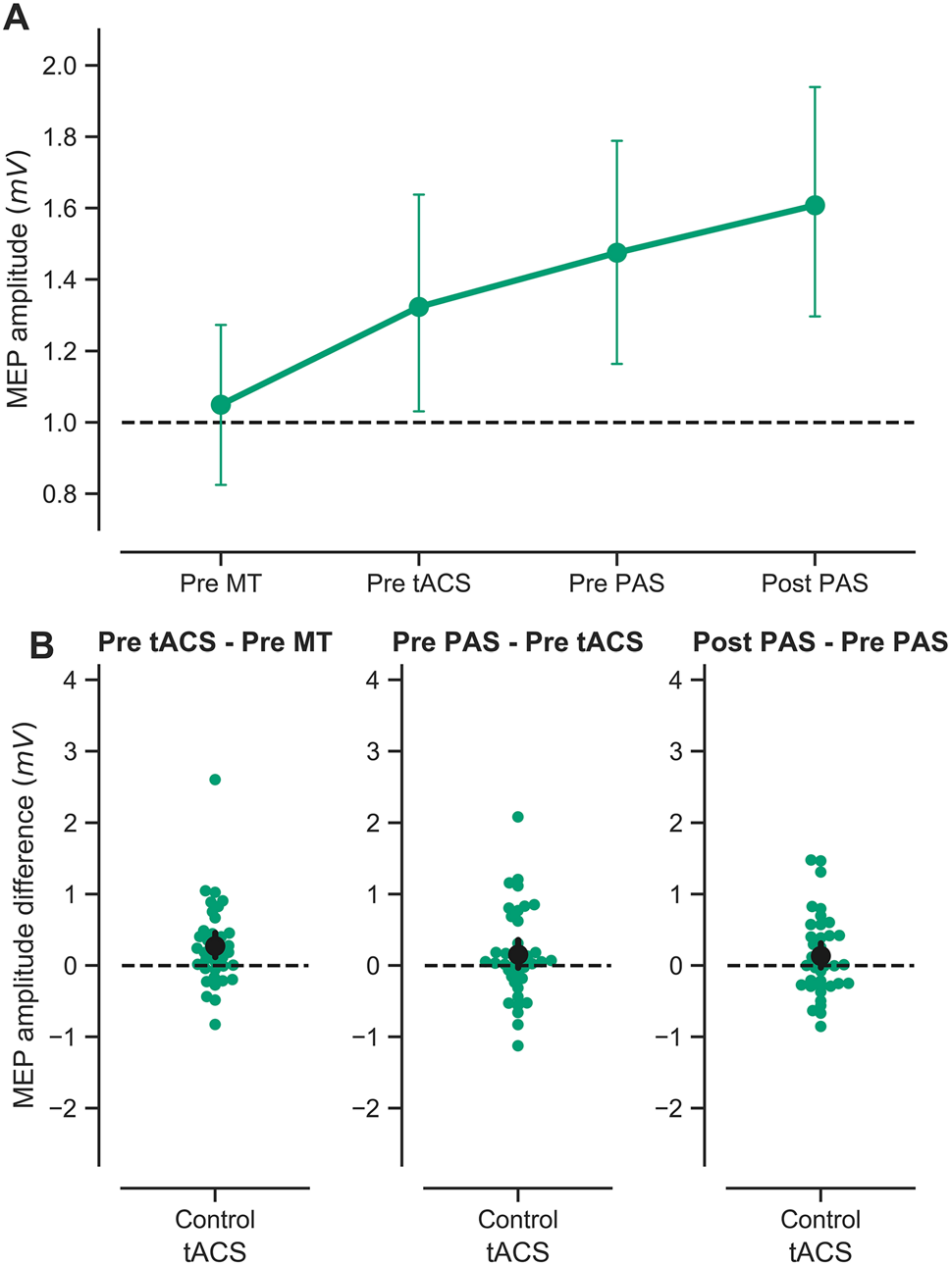
Effect of control tACS on MEP amplitude in response to successive plasticity paradigms. **A** Raw MEP amplitude (mV) for the four experimental time points: at baseline (Pre MT), after motor training/before tACS (Pre tACS), after tACS/before PAS (Pre PAS) and after PAS (Post PAS), in the control (green) tACS sessions. Symbols represent the mean; error bars denote 95% confidence intervals (CI, 1000 bootstrap iterations). Dotted line denotes baseline MEP amplitude. **B** Mean MEP amplitude difference over each intervention (Post – Pre, black symbol), with 95% CI (black bar) and individual observations (coloured symbols). Effect of 30 min of ballistic motor training (MT) on MEP amplitude (left). Effect of 18 min. of slow-oscillatory transcranial alternating current stimulation (tACS) (middle). Effect of paired associative stimulation (PAS_25_, excitatory protocol) on MEP amplitude (right). Dotted line denotes null difference between time points. *N* = 38

For the control tACS condition, both acceleration and MEPs were increased after motor training (Fig. 3A, B left panel). A Bayesian paired t-test on horizontal thumb acceleration revealed strong evidence that acceleration differed before and after training (BF_10_ = 6.27 × e^5^, Cohen’s *d* = 1.02). Similarly, there was moderate evidence that MEP amplitudes differed before and after training (BF_10_ = 6.73, Cohen’s *d* = 0.48). These results confirm that the initial plasticity paradigm (motor training by ballistic thumb abduction) was effective in improving performance and in inducing plasticity in the motor system.

*No evidence for modulation of MEPs immediately after tACS following motor training.* Next, we examined the effect of tACS on MEPs immediately after tACS, and prior to PAS. We hypothesised that in the sham tACS and control tACS conditions, MEPs would remain elevated relative to baseline, or increase further as a consequence of motor learning. We did not have a specific hypothesis concerning MEPs following active tACS, as slow oscillations could be hypothesised to reduce MEPs, or to modulate synaptic scaling without affecting MEP amplitude directly. Overall, there was little evidence of further change in MEP amplitudes after tACS (Figs. 2B, 3B, middle panels), consistent with previous research demonstrating that MEP increases induced by motor training persist for at least 30 min following training (Ziemann et al. 2004). For the sham and active tACS conditions, a Bayesian two-way repeated-measures ANOVA revealed moderate evidence against a main effect of time (BF_incl_ = 0.14, η_p_^2^ = 7.46 × e^−5^), anecdotal evidence against a main effect of tACS type (BF_incl_ = 0.37, η_p_^2^ = 0.039) and strong evidence against the interaction of time and tACS type (BF_incl_ = 0.1, η_p_^2^ = 0.118). For the control tACS condition, a Bayesian paired t-test before and after tACS provided anecdotal evidence that MEP amplitudes did not differ over time (BF_10_ = 0.47, Cohen’s *d* = 0.24).

*tACS following motor training does not enable subsequent PAS plasticity.* A prediction arising from the synaptic homeostasis hypothesis is that slow-wave oscillations “reset” synaptic connections back to a functional range, making them more receptive to subsequent plasticity paradigms. Thus, we predicted that the excitatory effect of PAS should manifest more robustly when preceded by active tACS (which should “unload” the targeted synapses) compared with sham tACS (where synaptic plasticity should have become relatively more saturated). In contrast, the control tACS montage should not “reset” synaptic connections in the network of interest and thus should not prevent interference of motor training with PAS. We found evidence suggesting that active tACS does not restore PAS effects after motor training (Fig. 2B right panel). Furthermore, results were equivocal with respect to the question of whether PAS modulates MEP amplitudes after sham tACS and control tACS (Figs. 2B, 3B right panels). For the sham and active tACS conditions, a Bayesian two-way repeated-measures ANOVA revealed moderate evidence against a main effect of time (BF_incl_ = 0.15, η_p_^2^ = 0.007), anecdotal evidence against a main effect of tACS (BF_incl_ = 0.43, η_p_^2^ = 0.042) and moderate evidence against the interaction of time and tACS type (BF_incl_ = 0.11, η_p_^2^ = 0.084). Post-hoc Bayesian paired *t*-tests revealed anecdotal and moderate evidence levels against an effect of PAS following sham tACS (BF_10_ = 0.88, Cohen’s *d* =  – 0.32) and active tACS (BF_10_ = 0.22, Cohen’s *d* = 0.1), respectively. For the control tACS condition, a Bayesian paired t-test revealed anecdotal evidence against the effect of time on MEP amplitudes (BF_10_ = 0.46, Cohen’s *d* = 0.24).

**Fig. 4:**
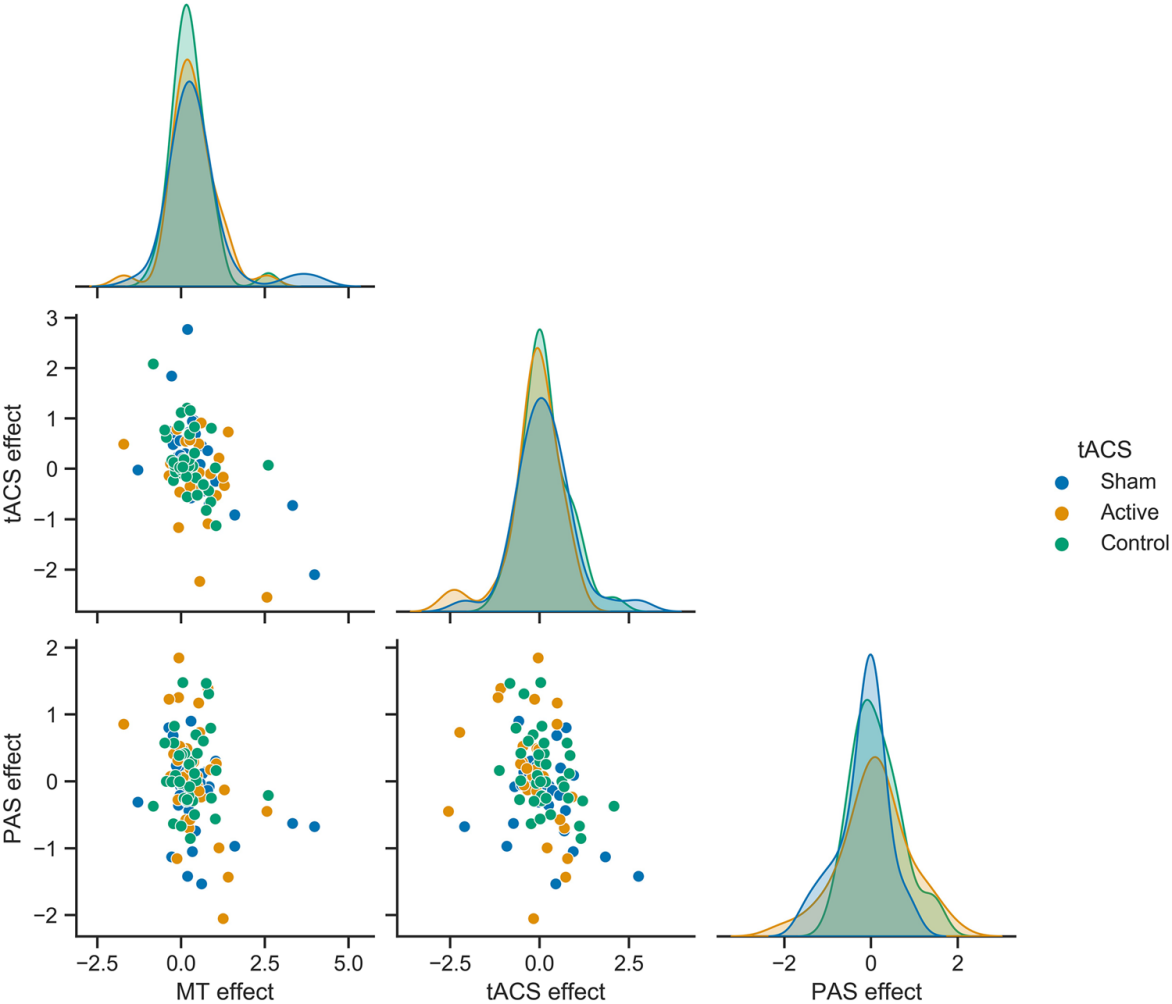
Relationship between the effects of successive plasticity interventions. Scatterplots comparing the effect of motor training (MT), slow-oscillatory transcranial alternating current stimulation (tACS), and paired associative stimulation (PAS), in the sham fronto-motor (blue), active fronto-motor (orange) and control posterior midline (green) tACS conditions. Results are expressed as the difference in MEP amplitude (mV) (Post – Pre) for the different interventions. Symbols represent individuals and shaded curves represent estimates of the probability density functions of the variables. Regions of overlap between the curves are shown in grey

Exploratory visualisation of the relationship between the effect of different interventions (expressed as the difference in MEP amplitudes Post – Pre intervention) did not reveal any trends in the data beyond those outlined above (Fig. 4). Data from the other two hand muscles, ADM and FDI, are presented in Supplementary Materials.

*Assessing PAS effects using an adjusted stimulation intensity.* In a subset of participants who received active and sham tACS (*N* = 29), intensity of stimulation was readjusted prior to PAS to elicit MEPs of similar amplitude to baseline. This new intensity was used to record MEPs before and after PAS to address potential concerns that PAS effects may be masked by ceiling effects. Results did not provide conclusive evidence that active tACS modulates PAS effects differently to sham tACS (Table 2). A Bayesian two-way repeated-measures ANOVA revealed moderate evidence in favour of a main effect of time (BF_incl_ = 3.62, η_p_^2^ = 0.251), anecdotal evidence against a main effect of tACS (BF_incl_ = 0.73, η_p_^2^ = 0.069) and anecdotal evidence against the interaction of time and tACS type (BF_incl_ = 0.49, η_p_^2^ = 0.024). Post-hoc Bayesian paired t-tests revealed anecdotal and moderate evidence for an effect of PAS following sham tACS (BF_10_ = 1.16, Cohen’s *d* = 0.38) and active tACS (BF_10_ = 3.53, Cohen’s *d* = 0.49), respectively. However, there was anecdotal evidence against a difference in MEP amplitudes between active and sham sessions after PAS (BF_10_ = 0.59, Cohen’s *d* = 0.29).

**Table 2:** MEP amplitude (mV), recorded at adjusted intensity. Mean ± SD

	*N*	Pre PAS	Post PAS
Active tACS	29	1.1 ± 0.95	1.37 ± 1.07
Sham tACS	29	0.99 ± 0.92	1.17 ± 1

**Fig. 5:**
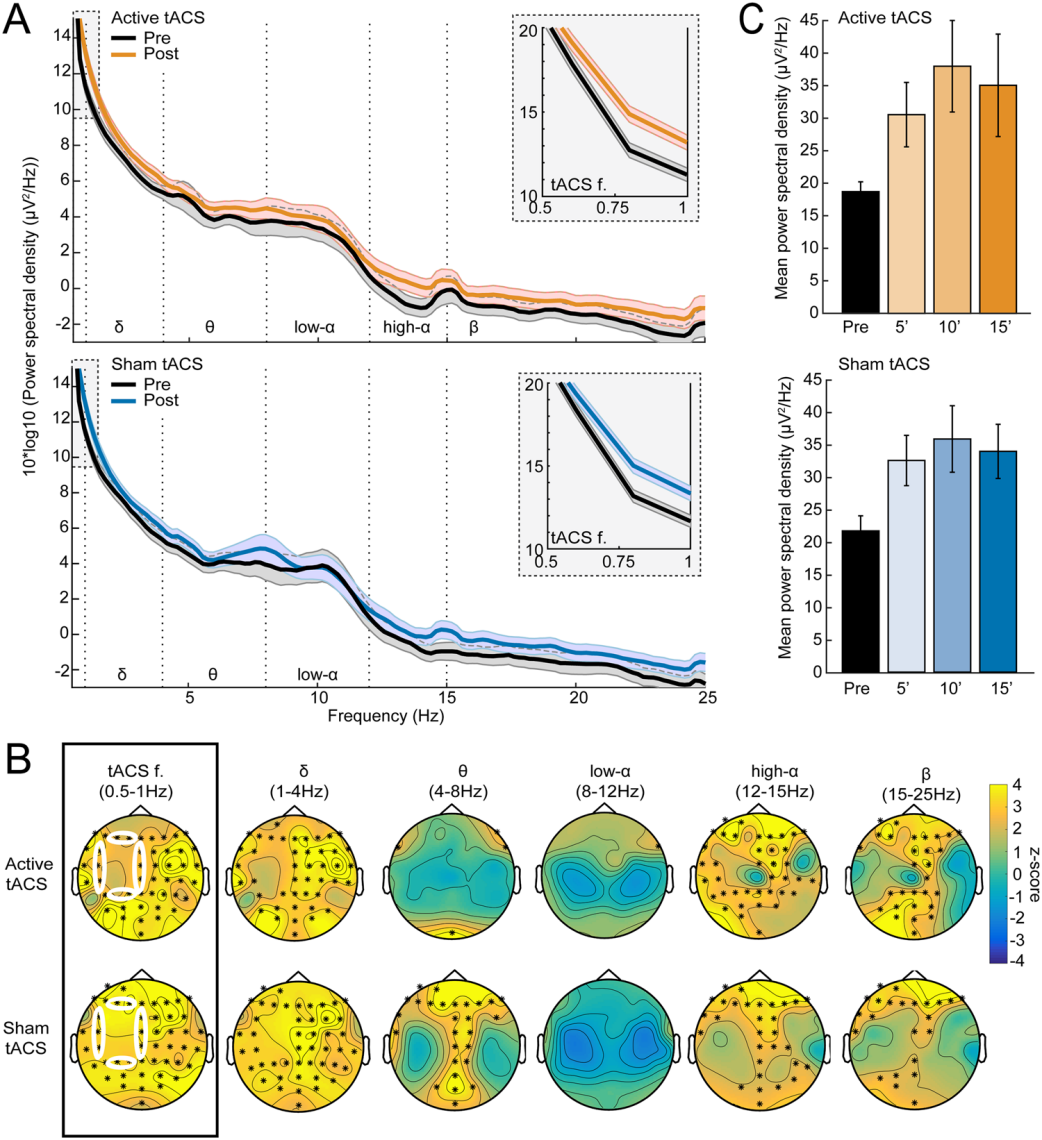
Off-line modulation of EEG power by tACS. **A** Average EEG power spectrum in the electrodes surrounding the motor tACS pad (10 pooled electrodes, see white circles and details in panel B), just before tACS (black curves) and immediately after each block of tACS (average of three 1-min segments, coloured curves: active tACS in orange, sham tACS in blue). The tACS-frequency band (0.5–1 Hz) is highlighted in grey and enlarged in the grey inset for readability. Error bars denote SEM, N = 25 participants. **B** Cluster-based permutation analysis contrasting power values before and after tACS (top row: active, bottom row: sham) in each of the 6 frequency bands of interest (tACS frequency, delta, theta, low alpha, high alpha and beta). Scalp maps display z-scores of the initial sample statistic and *denotes *p* < 0.01 for electrodes belonging to a significant cluster. The 10 electrodes surrounding the motor tACS pad (FCz, Cz, CPz, CP5, C5, FC5, F3, P3, F1, P1) are highlighted by white ellipses in the scalp maps of tACS frequency (black rectangle). **C** Mean power in the tACS-frequency band before, after 5 min, 10 min and 15 min of tACS, for active tACS (top, orange) and sham tACS (bottom, blue). Average across participants in the pooled electrodes surrounding the motor tACS pad. An ANOVA revealed a significant effect of time, but there was no effect of stimulation type or interaction between these factors. Errors bars denote SEM, *N* = 25 participants

*No evidence for specific entrainment of slow oscillations immediately after active tACS.* To investigate whether active tACS entrained brain oscillations at its frequency of delivery (0.75 Hz) or caused other modulations of oscillatory activity, we investigated changes in scalp EEG power immediately after each of the three blocks of 5 min of tACS, as compared to just before (Fig. 5A). A (non-Bayesian) cluster-based permutation test revealed no significant difference at baseline between sham and active tACS sessions. The same statistical approach uncovered a significant increase in power after both active (2 clusters: *p* = 0.001, cluster statistic = 320; *p* = 0.006, cluster statistic = 206) and sham (2 clusters: *p* = 0.001, cluster statistic = 413; *p* = 0.009, cluster statistic = 151) tACS separately (Fig. 5B). Following both sham and active stimulation, there was a widespread power increase in slow-oscillation (0.5-1 Hz) and delta (1–4 Hz) frequency bands, as well as a more spatially limited increase in high alpha (12–15 Hz) and beta (15–25 Hz) frequencies. Direct comparison of both raw power and decibel-change values from baseline revealed no significant difference between active and sham sessions post-tACS. Focussing on the tACS-frequency band, a two-way repeated-measures ANOVA of pooled power values in the electrodes surrounding the motor tACS pad revealed a significant effect of time (F(3,72) = 7.9, *p* = 0.0001), but not of tACS type (active or sham, F(1,24) = 0.008, *p* = 0.93), and there was no interaction between these factors (F(3,72) = 0.2, *p* = 0.89) (Fig. 5C). Thus, there was no evidence for a frequency-specific entrainment effect in the EEG data that outlasted the period of direct stimulation.

## Discussion

We set out to test whether a short period of tACS could attenuate the interference between two successive motor plasticity paradigms in awake humans. We found that motor training by repetitive, ballistic thumb movements induced an increase in MEPs. Importantly, we found moderate evidence against an effect of active tACS in restoring PAS plasticity, together with no evidence of lasting entrainment of slow oscillations in the EEG. This suggests that, under the conditions tested here, slow-oscillatory tACS does not modulate synaptic homeostasis in the motor system of awake humans.

As some properties of PAS are reminiscent of long-term potentiation (LTP) and long-term depression (LTD) properties in animal preparations (e.g., NMDA-receptor dependence, time-course, spatial specificity) (Stefan et al. 2000, 2002; Wolters et al. 2003), PAS effects in humans have been labelled as ‘LTP/LTD-like’ plasticity (Stefan et al. 2000; Wolters et al. 2003; Ziemann et al. 2004) and interpreted as being analogous to neuronal LTP/LTD in animal experiments. The fact that PAS effects are impeded or even reversed by prior motor practice suggests that the initial motor practice triggered LTP-like plasticity, leading to modification of the LTP/LTD induction threshold, and tipping the balance in favour of LTD-like plasticity induction (Ziemann et al. 2004). Here, as in the seminal report (Ziemann et al. 2004), we showed attenuation but not reversal of ‘excitatory’ PAS effects by motor practice in the sham and control conditions, which is compatible with the notions of saturation of LTP-like processes and modification of the LTP/LTD threshold. Importantly, following active tACS, PAS did not lead to an enhancement of MEPs that was substantially different from sham. This could have occurred for a number of reasons, which are discussed below.

While there is no consensus regarding the mechanisms of action of tACS, recent literature has focussed on detecting local, long-lasting increases in oscillatory power at the stimulation frequency, which could be explained in terms of entrainment of cortical oscillatory activity (Ozen et al. 2010; Ali et al. 2013; Helfrich et al. 2014; Alagapan et al. 2016, although see Vossen et al. 2015 for a network plasticity account). More specifically, with regard to slow oscillations, a number of studies using scalp EEG recordings in humans have reported locally increased power in the low-frequency band immediately following slow-oscillatory tDCS to frontal cortex (Marshall et al. 2006; Kirov et al. 2009; Antonenko et al. 2013; Westerberg et al. 2015; Paßmann et al. 2016; Ladenbauer et al. 2016, 2017). In contrast, the current study found no evidence for a specific power increase in the low-frequency band immediately following active tACS.

This apparent discrepancy with previous studies is likely attributable to several factors. First, all but one previous study delivered slow-oscillatory tDCS during sleep-either a nap or a full-night’s sleep (Marshall et al. 2006; Antonenko et al. 2013; Westerberg et al. 2015; Paßmann et al. 2016; Ladenbauer et al. 2016, 2017), when cortical slow oscillations are expected to occur endogenously (Steriade et al. 1993). It is possible that to achieve modulation of slow oscillations via tACS, the network needs to be in a state that enables generation of slow oscillations, or that such oscillations need to be present at the time of stimulation. Consistent with this hypothesis, both modelling and animal stimulation experiments have shown that weak oscillatory currents are most effective at modulating endogenous oscillations in cortical networks (Schmidt et al. 2014). Second, most previous studies employed a stimulation waveform containing a direct current (DC) component (slow-oscillatory tDCS), as opposed to the pure tACS used here. Even though such an explanation would render frequency-specific power modulations harder to account for, it is conceivable that the EEG after-effects described in previous literature resulted from the DC component rather than the oscillating component of slow-oscillatory tDCS. Third, in previous studies, stimulation was applied to the frontal cortex, which has been shown to be a prominent source of slow oscillations (Massimini 2004; Sheroziya and Timofeev 2014). It is conceivable that tACS applied through a classic ‘motor’ parieto-frontal montage does not modulate low-frequency oscillations because it targets a fundamentally different functional network that is less reliant on slow oscillations to achieve consolidation of memories. However, studies have highlighted the existence of ‘local’ slow oscillations, even in motor cortex (Huber et al. 2013; Fattinger et al. 2017). Fourth, as our evaluation of tACS effects on EEG took place immediately after an episode of motor learning, it is possible that learning-related EEG modulations in both the sham and active tACS conditions masked the effects of tACS alone. Finally, it is important to note that a number of studies using variations of slow-oscillatory tDCS/tACS failed to find lasting modulation of low-frequency oscillations (Eggert et al. 2013; Sahlem et al. 2015; D’Atri et al. 2016; Bueno-Lopez et al. 2019). Most notably, Lafon et al. (2017) reported a lack of entrainment to slow-wave tACS, as measured by implanted electrodes in human epileptic patients.

Importantly, the EEG results reported here do not speak to the likelihood of other mechanisms of action of tACS (for a review, see (Liu et al. 2018)), such as modulation of neuronal activity and excitability *during* tACS. We recorded EEG during tACS, but did not analyse the resulting data, as the physiological electric signal is strongly distorted by the tACS-injected current, and satisfactory correction of these distortions is still very much under debate (see e.g., (Noury and Siegel 2017; Neuling et al. 2017; Kasten and Herrmann 2019)). Other hypothesised neuronal mechanisms of action (e.g. Asamoah et al. 2019) are currently the subject of intense discussion, with some authors suggesting that the stimulation intensities currently used might not be sufficient to cause measurable modulation of neuronal activity (Lafon et al. 2017; Vöröslakos et al. 2018), but see (Krause et al. 2019). This raises the question of whether tACS as applied here was effective in reaching the brain and in altering brain activity. Altogether, converging evidence points to the fact that low electric fields (< 1 V/m, and as low as ~ 0.2 V/m) can modulate neuronal membrane potential and consequently bias the timing of ongoing spiking activity without necessarily affecting firing rate per se (e.g. Reato et al. 2013; Huang et al. 2021; Tran et al. 2022). Importantly, the modelled electric field resulting from our montage reaches up to 0.228 V/m, which is in the range for effective neuronal activity modulation.

The current study has a number of limitations. First, given PAS can be a variable protocol, we cannot fully exclude the interpretation that our results were due to PAS being ineffective. We sought robust conditions for the PAS protocol by: (1) testing relatively large numbers of participants (*N* = 36 and 38), giving a high likelihood of detecting true effects, (2) using a well-established PAS protocol (Ziemann et al. 2004), (3) controlling for attentional variability, which has been shown to impact PAS effects (Kamke et al. 2014) and (4) assessing PAS effects with adjusted intensity to prevent ceiling effects in the main experiment. However, we did not provide a separate replication of the efficacy of PAS on its own. In a similar way, we did not test the effect of tACS alone. Additional experiments assessing the effect of tACS alone and tACS following motor training (with no subsequent PAS) on MEP amplitude could help ascertain whether tACS has MEP-modulating effects on its own, and if so, whether it interacts with motor training in a homeostatic way. However, previous research has failed to show any effects of slow-oscillatory tACS on motor cortical excitability (Antal et al. 2008). Another limitation is that we did not re-test ballistic thumb movement acceleration after tACS or after PAS. Consequently, we are not able to ascertain whether the levels of performance were maintained throughout the session. Our motivation for not re-assessing motor performance was two-fold. First, we wished to avoid any interference that might arise from having participants perform the motor task again, as it has been suggested that motor activity can reverse or abolish the expression of rTMS-induced plasticity (see e.g. Iezzi et al. 2008 for theta-burst stimulation-induced changes). While this is not specific to PAS, it led us to decide against asking participants to perform ballistic thumb movements after the initial motor training, over concern that further motor activity would interfere with tACS or PAS. Second, additional thumb acceleration measurements would have added time to an already lengthy experiment, which might have adversely impacted participant arousal level or motivation.

In conclusion, we interleaved tACS between two interventions known to induce plasticity in human motor cortex, and which have been shown previously to interfere with each other. We found evidence against a modulation of cortico-spinal excitability by PAS after active tACS, which suggests that under the conditions used here, tACS does not modulate plasticity interactions in the motor cortex.

## Supplementary Information

Below is the link to the electronic supplementary material.

Supplementary file1 (DOCX 153 KB)

## Data Availability

The datasets generated during and/or analysed during the current study are available from the corresponding author on reasonable request.
