# Supplementary material for: Slow-oscillatory tACS does not modulate human motor cortical response to repeated plasticity paradigms
Source: Exp Brain Res. 2022 Sep 29;240(11):2965–79. doi: 10.1007/s00221-022-06462-z (PMC9587974; doi:10.1007/s00221-022-06462-z)
Supplement: Supplementary file 1 — Supplementary file1 (DOCX 153 KB) [file 221_2022_6462_MOESM1_ESM.docx]

**Supplementary Materials**

**Rationale and methods:** In order to test the spatial specificity of any plasticity effects, MEPs were recorded from two hand muscles that were not recruited by motor training and that were not specifically targeted by PAS: *abductor digiti minimi* (ADM) and first dorsal interosseous (FDI). MEPs were analysed in the same way as data from APB (see main manuscript). Final participant numbers are lower due to missing data.

**Results:** Overall, and as expected, plasticity interventions had little effect on MEPs from ADM and FDI.

For ADM (**Fig. S1**), a Bayesian two-way repeated-measures ANOVA before and after motor training revealed moderate evidence against a main effect of time (BF_incl_ = 0.176), with moderate evidence against a main effect of tACS type (BF_incl_ = 0.138) and strong evidence against their interaction (BF_incl_ = 0.041). Similarly, focusing on the effect of tACS on MEPs, there was moderate evidence against a main effect of time (BF_incl_ = 0.174), with moderate evidence against a main effect of tACS type (BF_incl_ = 0.229) and strong evidence against their interaction (BF_incl_ = 0.05). Finally, examining the effect of PAS on MEPs, there was moderate evidence against a main effect of time (BF_incl_ = 0.143), with moderate evidence against a main effect of tACS type (BF_incl_ = 0.218) and strong evidence against their interaction (BF_incl_ = 0.042).


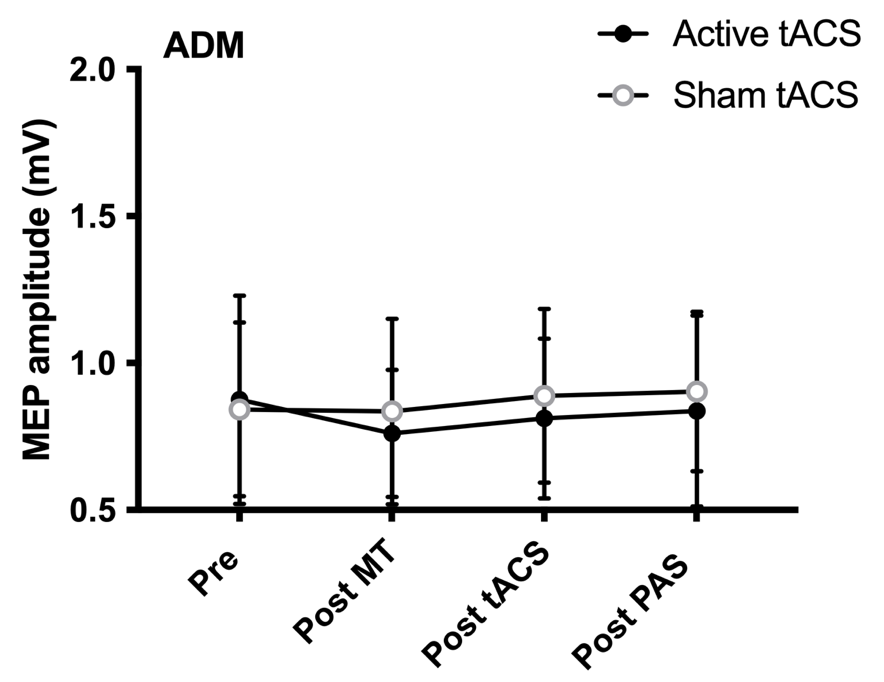


**Figure S1.** *Abductor digiti minimi* (ADM) raw MEP amplitude (mV) at four experimental time-points: at baseline (Pre), after motor training/before tACS (Post MT), after tACS/before PAS (Post tACS) and after PAS (Post PAS), in the sham (white) and active (black) tACS sessions. Symbols represent the mean; error bars denote 95% confidence intervals. N=31.

For FDI (**Fig. S2**), a Bayesian two-way repeated-measures ANOVA before and after motor training revealed moderate evidence against a main effect of time (BF_incl_ = 0.21), with anecdotal evidence against a main effect of tACS type (BF_incl_ = 0.868) and moderate evidence against their interaction (BF_incl_ = 0.127). Similarly, focusing on the effect of tACS on MEPs, there was moderate evidence against a main effect of time (BF_incl_ = 0.228), with anecdotal evidence against a main effect of tACS type (BF_incl_ = 0.892) and moderate evidence against their interaction (BF_incl_ = 0.13). Finally, examining the effect of PAS on MEPs, there was moderate evidence for a main effect of time (BF_incl_ = 2.39), with strong evidence for a main effect of tACS type (BF_incl_ = 11.9) and anecdotal evidence against their interaction (BF_incl_ = 0.903). Post-hoc Bayesian paired t-tests revealed anecdotal and moderate evidence against an effect of PAS following sham tACS (BF_10_ = 0.362) and active tACS (BF_10_ = 0.202), respectively.


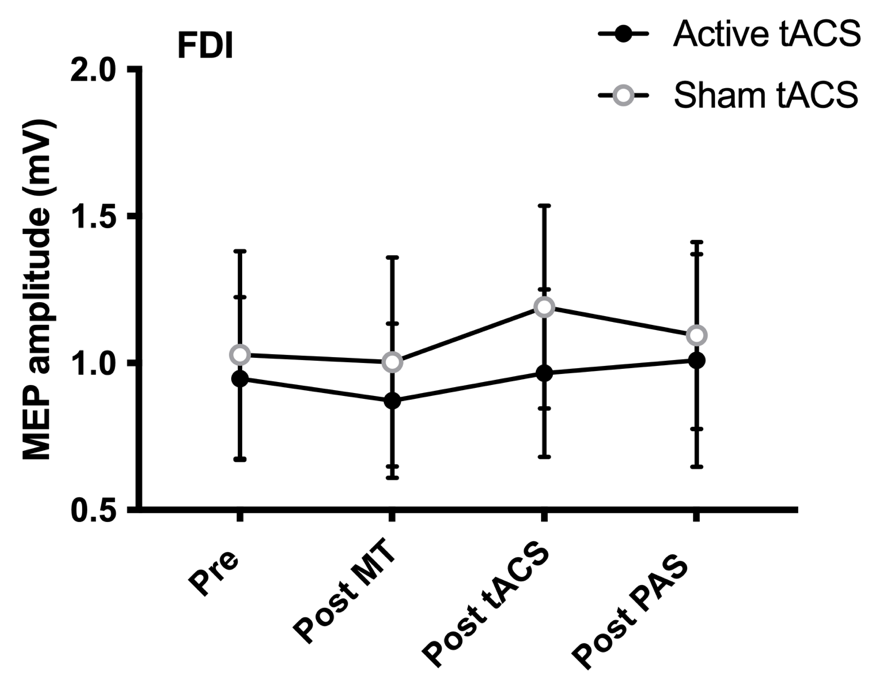


**Figure S2.** First dorsal interosseous (FDI) raw MEP amplitude (mV) at four experimental time-points: at baseline (Pre), after motor training/before tACS (Post MT), after tACS/before PAS (Post tACS) and after PAS (Post PAS), in the sham (white) and active (black) tACS sessions. Symbols represent the mean; error bars denote 95% confidence intervals. N=34.
